# Denoising approach with deep learning-based reconstruction for neuromelanin-sensitive MRI: image quality and diagnostic performance

**DOI:** 10.1007/s11604-023-01452-9

**Published:** 2023-05-31

**Authors:** Sonoko Oshima, Yasutaka Fushimi, Kanae Kawai Miyake, Satoshi Nakajima, Akihiko Sakata, Sachi Okuchi, Takuya Hinoda, Sayo Otani, Hitomi Numamoto, Koji Fujimoto, Atsushi Shima, Masahito Nambu, Nobukatsu Sawamoto, Ryosuke Takahashi, Kentaro Ueno, Tsuneo Saga, Yuji Nakamoto

**Affiliations:** 1 Department of Diagnostic Imaging and Nuclear Medicine, Graduate School of Medicine, Kyoto University, 54 Shogoin Kawahara-Cho, Sakyo-Ku, Kyoto, 606-8507 Japan; 2 Department of Advanced Medical Imaging Research, Graduate School of Medicine, Kyoto University, 54 Shogoin Kawahara-Cho, Sakyo-Ku, Kyoto, 606-8507 Japan; 3 Department of Real World Data Research and Development, Graduate School of Medicine, Kyoto University, 54 Shogoin Kawahara-Cho, Sakyo-Ku, Kyoto, 606-8507 Japan; 4 Department of Regenerative Systems Neuroscience, Human Brain Research Center, Graduate School of Medicine, Kyoto University, 54 Shogoin Kawahara-Cho, Sakyo-Ku, Kyoto, 606-8507 Japan; 5 MRI Systems Division, Canon Medical Systems Corporation, 1385 Shimoishigami, Otawara-Shi, Tochigi, 324-0036 Japan; 6 Department of Human Health Sciences, Graduate School of Medicine, Kyoto University, 53 Shogoin Kawahara-Cho, Sakyo-Ku, Kyoto, 606-8507 Japan; 7 Department of Neurology, Graduate School of Medicine, Kyoto University, 54 Shogoin Kawahara-Cho, Sakyo-Ku, Kyoto, 606-8507 Japan; 8 Department of Biomedical Statistics and Bioinformatics, Graduate School of Medicine, Kyoto University, 54 Shogoin Kawahara-Cho, Sakyo-Ku, Kyoto, 606-8507 Japan

**Keywords:** Deep learning, Denoising, Neuromelanin, Magnetic resonance imaging, Parkinson’s disease

## Abstract

**Purpose:**

Neuromelanin-sensitive MRI (NM-MRI) has proven useful for diagnosing Parkinson’s disease (PD) by showing reduced signals in the substantia nigra (SN) and locus coeruleus (LC), but requires a long scan time. The aim of this study was to assess the image quality and diagnostic performance of NM-MRI with a shortened scan time using a denoising approach with deep learning-based reconstruction (dDLR).

**Materials and methods:**

We enrolled 22 healthy volunteers, 22 non-PD patients and 22 patients with PD who underwent NM-MRI, and performed manual ROI-based analysis. Signal-to-noise ratio (SNR) and contrast-to-noise ratio (CNR) in ten healthy volunteers were compared among images with a number of excitations (NEX) of 1 (NEX1), NEX1 images with dDLR (NEX1 + dDLR) and 5-NEX images (NEX5). Acquisition times for NEX1 and NEX5 were 3 min 12 s and 15 min 58 s, respectively. Diagnostic performances using the contrast ratio (CR) of the SN (CR_SN) and LC (CR_LC) and those by visual assessment for differentiating PD from non-PD were also compared between NEX1 and NEX1 + dDLR.

**Results:**

Image quality analyses revealed that SNRs and CNRs of the SN and LC in NEX1 + dDLR were significantly higher than in NEX1, and comparable to those in NEX5. In diagnostic performance analysis, areas under the receiver operating characteristic curve (AUC) using CR_SN and CR_LC of NEX1 + dDLR were 0.87 and 0.75, respectively, which had no significant difference with those of NEX1. Visual assessment showed improvement of diagnostic performance by applying dDLR.

**Conclusion:**

Image quality for NEX1 + dDLR was comparable to that of NEX5. dDLR has the potential to reduce scan time of NM-MRI without degrading image quality. Both 1-NEX NM-MRI with and without dDLR showed high AUCs for diagnosing PD by CR. The results of visual assessment suggest advantages of dDLR. Further tuning of dDLR would be expected to provide clinical merits in diagnosing PD.

**Supplementary Information:**

The online version contains supplementary material available at 10.1007/s11604-023-01452-9.

## Introduction

Parkinson’s disease (PD) is a neurodegenerative disorder involving progressive loss of dopaminergic neurons in the substantia nigra (SN) and noradrenergic neurons in the locus coeruleus (LC), both of which contain pigments called neuromelanin [1, 2]. Neuromelanin is a strong chelator of heavy metals, particularly iron, and plays important roles in protecting against neurotoxicity caused by free iron [3, 4]. Symptoms of PD are thought to appear after 50–60% of dopamine neurons have degenerated, and the presymptomatic phase often spans more than 20 years [5, 6].

Given this background, neuromelanin-sensitive MRI (NM-MRI) methods have been investigated for the early diagnosis of PD [7–19]. Neuromelanin-iron complexes have T1-shortening effects, and magnetization transfer (MT) effects also contribute to the signal contrast by suppressing signals from background brain parenchyma [7–15, 17–20]. Previous NM-MRI studies have shown decreased signal intensity in the SN of patients with PD [7–19]. However, NM-MRI requires a relatively long scan time of 7–10 min, because a high resolution with an adequate signal-to noise ratio (SNR) is required due to the small sizes of the SN and LC, and NM-MRI is usually acquired using a number of excitations (NEX) greater than 1 [7–10, 12–15, 19]. This long scan time causes difficulty in obtaining sufficient image quality from PD patients with tremor or involuntary movements. A smaller NEX would reduce the scan time, but at the expense of reducing SNR. Although short-scan MR techniques have been tried for NM-MRI (e.g., NM-MRI using a chemical shift selective pulse instead of an MT pulse with scan time of 3 min 20 s and lower image resolution), the diagnostic performance for PD has not been evaluated. [21]. A few reports have described the application of parallel imaging to NM-MRI, and none appear to have used compressed sensing [22, 23].

A denoising approach with deep learning-based reconstruction (dDLR) has been applied for MRI recently [24–27]. The dDLR is trained using vast amounts of high-quality image data, and makes use of a deep learning neural network to remove image noise and produce clear images in clinical practice. We hypothesized that short-scan time NM-MRI of sufficient image quality would be achievable by applying dDLR to NM-MRI with a fewer NEX. To shorten the scan time as much as possible, we used NEX-1 NM-MRI as source images. To the best of our knowledge, no previous studies have examined the image quality or diagnostic accuracy of NM-MRI with dDLR.

The purposes of this study were thus: (1) to compare image quality between NEX-1 NM-MRI without dDLR, NEX-1 NM-MRI with dDLR, and a reference standard of NEX-5 NM-MRI; and (2) to compare the diagnostic capability of NEX-1 NM-MRI with and without dDLR for differentiating patients with PD from non-PD patients.

## Materials and methods

### Study population

**Fig. 1:**
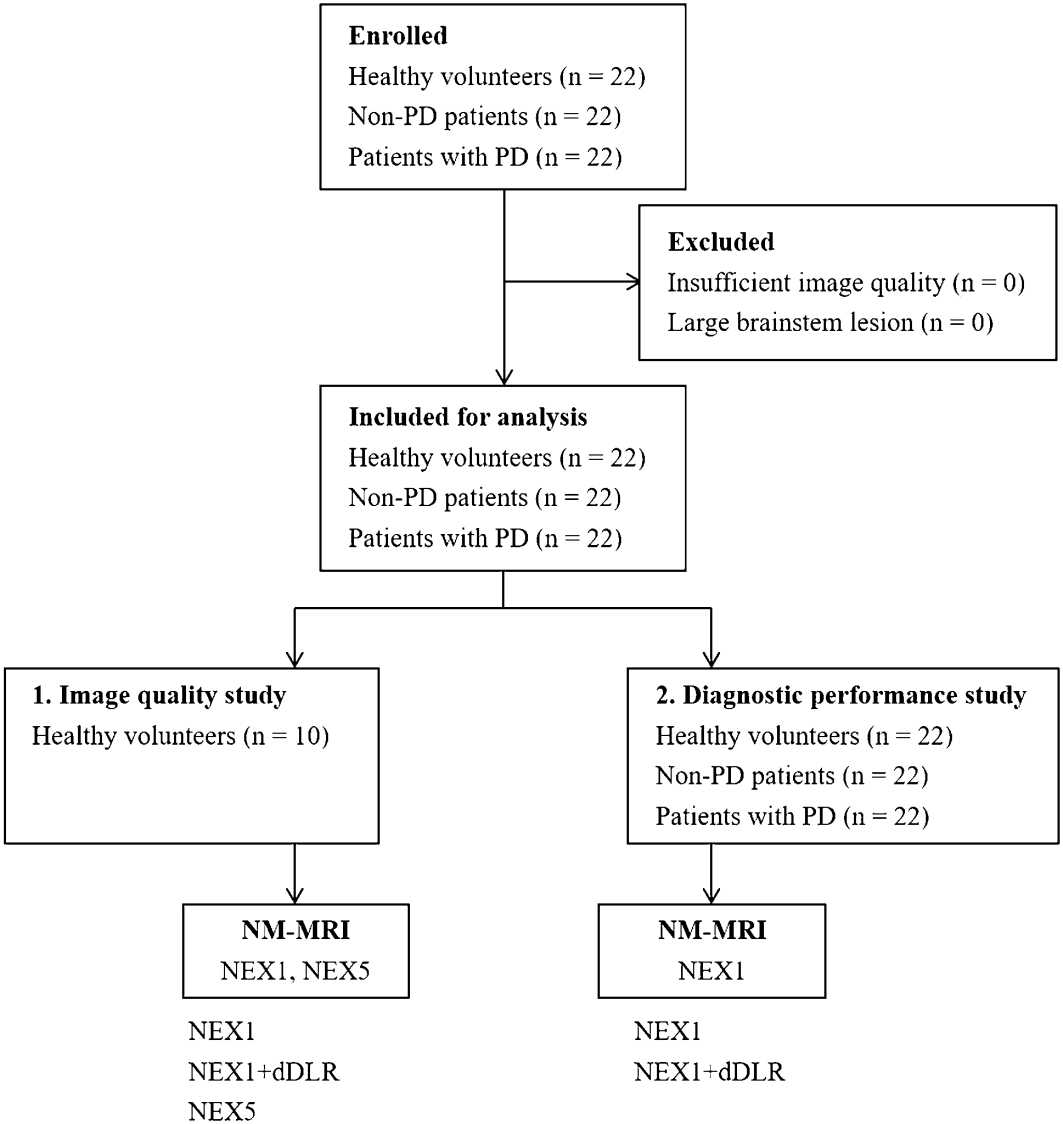
Flowchart of study participants and image analysis. *PD* Parkinson’s disease, *NEX* number of excitations, *dDLR* denoising approach with deep learning-based reconstruction

This prospective study was approved by the institutional ethics committee. Written informed consent was obtained from both healthy volunteers and patients prior to enrolment. For the image quality study, we prospectively enrolled 22 healthy volunteers. We recruited relatively young volunteers, because we needed them to stay still for about 16 min to acquire images from NEX-5 NM-MRI. For the diagnostic performance study, we enrolled 22 patients with PD who agreed to participate in this study, all of whom fulfilled the Movement Disorder Society PD Criteria for the diagnosis of PD [28], and 22 age- and sex-matched non-PD patients. Underlying diseases in the non-PD patients were brain aneurysm (*n* = 13), old brain infarction or ischemic change (*n* = 6) and cerebral artery stenosis (*n* = 3). All the lesions were located outside of the brainstem and no apparent brainstem abnormalities associated with old brain infarction such as Wallerian degeneration were observed. All participants underwent NM-MRI at our hospital between September 2019 and March 2021. No participants were excluded due to insufficient image quality or large brainstem lesions. Figure 1 summarizes the participant inclusion process and image analysis.

### Image acquisition

We acquired images from NM-MRI using a 2-dimensional gradient echo (2D-GRE) pulse sequence with MT contrast (MTC) preparation on a 3-T scanner (Vantage Centurian; Canon Medical Systems, Otawara, Japan) with a 32-channel head coil. 1-NEX NM-MRI was acquired from all healthy volunteers, and 5-NEX NM-MRI was acquired from 10 out of 22 volunteers (Fig. 1). For patients with PD, only 1-NEX NM-MRI was performed in this study. Brain MRI for screening had been finished on another day, and no specific abnormalities were found. For non-PD patients, 1-NEX NM-MRI and the other clinical brain scans were performed. Scan parameters were as follows: repetition time /echo time, 460/2.7 ms; 15 slices; slice thickness, 3 mm with no gap; field of view, 230 × 230 mm; matrix, 416 × 416; in-plane resolution, 0.55 × 0.55 mm; flip angle, 40°; bandwidth, 244.1 Hz/pixel; MTC pulses, 300°; off-resonance, 1.2 kHz; and acquisition time, 3 min 12 s for the 1-NEX scan and 15 min 58 s for the 5-NEX scan. No parallel imaging was applied.

### Post-imaging procedure

Denoising was applied to 1-NEX images (NEX1) using a commercially available deep learning-based reconstruction algorithm (Advanced intelligent Clear-IQ Engine [AiCE]; Canon Medical Systems) to create NEX1 + dDLR. AiCE is a whole reconstruction pipeline from raw complex data to final image generation. The complex images are processed for denoising in this pipeline which incorporates convolutional neural network. Its details were described in the previous literature [24]. Online Resource 1 displays its architecture. CNN architecture consists of multiple layers: the feature extraction, feature conversion and image generation layers. In the feature extraction layer, the input noisy image is convolved by the 7 × 7 discrete cosine transformation to derive 49 components, which is divided into 48 high-frequency components and a zero-frequency component. A soft-shrinkage activation function is applied to 48 high-frequency components. Next, the 48 high-frequency components undergo repeated 3 × 3 convolution and soft shrinkage in the feature conversion layers. Finally, in the image generation layer, the denoised output image is generated by the 7 × 7 inverse discrete cosine transform convolution of both the output data from the feature conversion layers and the bypassed zero-frequency component. The soft-shrinkage activation function enables adaptive noise removal using a threshold calculated from the noise level and a coefficient. Thus, there are two parameters to be trained: the 3 × 3 convolution kernels in the feature conversion layer and the coefficient of the soft-shrinkage activation function in the feature extraction and feature conversion layers. These parameters have been determined to minimize the differences between the training data and the output denoised image through the training process of AiCE.

### Image analysis

All images were analyzed using ImageJ software (National Institutes of Health) as the consensus decisions of 2 board-certified radiologists (S.O. and Y.F. with 9 and 22 years of experience in neuroradiology, respectively). Regions of interest (ROIs) of the SN, decussation of superior cerebellar peduncle (SCP), LC and pons were manually placed on the slice where the SN or LC was most clearly delineated (Online Resource 2). As for the ROIs of the SN, three circles were placed for right and left, respectively, and the signal intensities of the six ROIs were averaged [29]. The SCP and pons were used as background areas for the SN and LC, respectively. The shape and size of ROIs were the same in all images.

1. Image quality
  Image quality was assessed quantitatively and qualitatively using images from 10 healthy volunteers and from 22 patients with PD.
  For quantitative assessment, we calculated SNR of the SCP (SNR_SCP), SNR of the pons (SNR_pons), contrast-to-noise ratio (CNR) between SN and background SCP (CNR_SN) and CNR between LC and background pons (CNR_LC). We measured signal intensity (SI) in each ROI (SI_SCP_, SI_pons_, SI_SN_, and SI_LC_). SNR and CNR were defined as follows [30]:
  SNR_SCP = mean SI_SCP_ / SD of SI_SCP_,
  SNR_pons = mean SI_pons_ / SD of SI_pons_,
  CNR_SN = (mean SI_SN_ − mean SI_SCP_) / SD of SI_SCP_ and
  CNR_LC = (mean SI_LC_ − mean SI_pons_) / SD of SI_pons_, where SD is the standard deviation.
  For qualitative assessment of image quality, three neuroradiologists (S.N., S.O. and S.O. with 15, 13 and 10 years of experience in neuroradiology, respectively) visually evaluated overall image quality, artifacts, structural conspicuity and noise of the images at the SN and LC level by consensus using a 5-point Likert scale. The criteria for image assessment on the 5-point Likert scale are presented in Online Resource 3.
2. Diagnostic performance by contrast ratio
  To assess diagnostic performance, we calculated contrast ratios (CRs) of the SN and LC using images from 22 non-PD patients, 22 patients with PD and 22 healthy volunteers. CRs were defined as follows:
  CR_SN = mean SI_SN_ / mean SI_SCP_ and
  CR_LC = mean SI_LC_ / mean SI_pons_
3. Diagnostic performance by visual assessment
  Three neuroradiologists (S.N., S.O. and S.O. with 15, 13 and 10 years of experience in neuroradiology, respectively) visually assessed NEX1 and NEX1 + dDLR images at the level of the SN and LC, respectively, to differentiate PD from non-PD. Raters selected “PD”, “non-PD” and “difficult to diagnose” in accordance with the following criteria. As for SN, raters focused on whether the lateral part of SN is conspicuous or obscure. As for LC, raters focused on whether bilateral high intensities suggesting LC are well defined or not. If it was difficult to determine, “difficult to diagnose” was selected.

### Statistical analysis

For quantitative image quality analysis, SNRs and CNRs were compared among NEX1, NEX1 + dDLR and 5-NEX images without dDLR (NEX5) using analysis of variance (ANOVA) with Bonferroni correction for healthy volunteers, and among NEX1 and NEX1 + dDLR using paired t-test for patients with PD. For qualitative analysis, each qualitative index for the three types of images was compared using the Wilcoxon signed-rank test.

For diagnostic performance analysis by contrast ratio, we evaluated difference in CR_SN and CR_LC between age- and sex-matched non-PD and PD groups using Student’s *t*-test. We then performed receiver operating characteristic curve analyses for differentiating patients with PD from non-PD patients and compared the area under the receiver operating characteristic curve (AUC) between NEX1 and NEX1 + dDLR images using the DeLong test. In addition, differences in CR_SN and CR_LC between healthy and PD groups were assessed and receiver operating characteristic curve analyses were performed.

For diagnostic performance analysis by visual assessment, accuracy was calculated by dividing the number of cases with correct diagnosis by the total number of cases.

MedCalc version 20.009 software (MedCalc Software, Ostend Belgium) was used for statistical analyses, with differences of *p* < 0.05 considered significant.

## Results

The characteristics of participants are listed in Table 1. A total of 66 participants (age range, 26–86 years; mean ± SD age, 56.6 ± 17.0 years; 45 women) were enrolled including 22 healthy volunteers (age range, 26–54 years; mean age, 36.1 ± 7.8 years; 17 women), 22 patients with PD (age range, 58–86 years; mean age, 66.7 ± 9.6 years; 14 women) and age- and sex-matched 22 non-PD patients (age range, 49–82 years; mean age, 67.0 ± 9.1 years; 14 women).

**Table 1:** Characteristics of volunteers and patients

	Healthy volunteers	Non-PD patients	Patients with PD	*p* value**	*p* value***
Sex (Female/Male)	17/5	14/8	14/8	0.32	1
Age* [year]					
Total	36.1 ± 7.8 (26–54)	68.0 ± 11.9 (49–82)	66.7 ± 9.6 (53–86)	< 0.001	0.94
Female	36.5 ± 8.5 (28–54)	65.3 ± 9.1 (49–82)	68.0 ± 9.8 (53–86)	< 0.001	0.98
Male	34.0 ± 5.2 (26–40)	67.9 ± 9.1 (54–81)	64.5 ± 8.6 (55–83)	< 0.001	0.88
HY stage, medication on/off	–	–	2 (1–3)/3 (2–5)	–	–
UPDRS part III, medication on/off	*–*	*–*	24.5 (5–45)/44 (15–67)	–	–
Disease duration [year]	–	–	8 (1–30)	–	–

*Mean age ± standard deviation (years), with range shown in parentheses

**Healthy volunteers vs patients with PD

***Non-PD patients vs patients with PD

*PD* Parkinson’s disease, *HY* Hoehn and Yahr, *UPDRS* Unified Parkinson’s disease rating scale

**Fig. 2:**
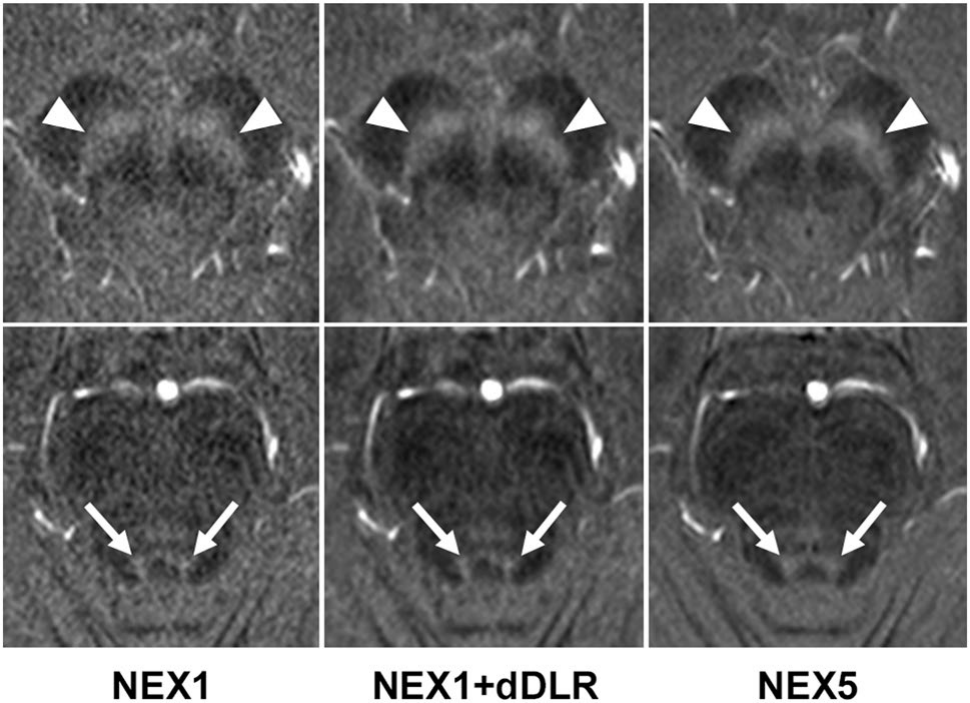
Images of the SN (arrowheads in the upper row) and LC (arrows in the lower row) from NEX1, NEX1 + dDLR and NEX5 for a 30-year-old healthy female participant. NEX, number of excitations; dDLR, denoising approach with deep learning-based reconstruction

**Fig. 3:**
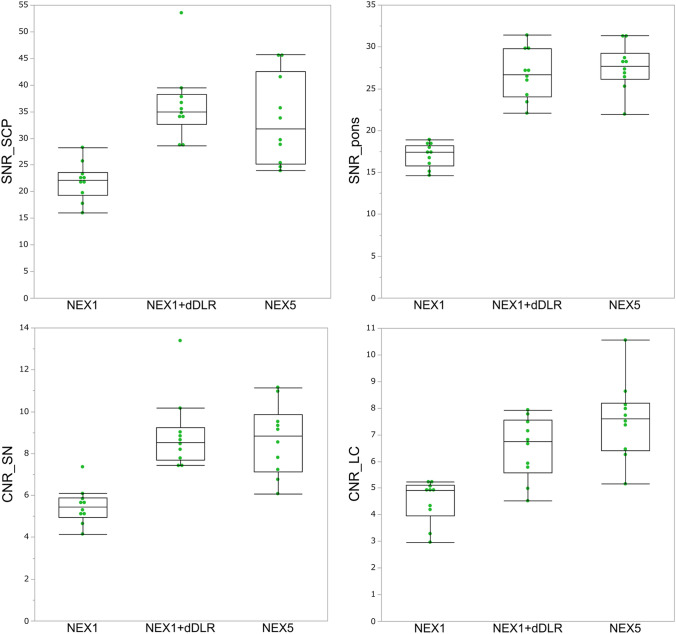
Box-and-whisker plots and scatter plots for SNR and CNR from NEX1, NEX1 + dDLR and NEX5 images of healthy volunteers. SNR and CNR from NEX1 + dDLR were significantly higher than those from NEX1 and showed no significant difference from those of NEX5. *SNR* signal-to-noise ratio; *CNR* contrast-to-noise ratio, *NEX* number of excitations, *dDLR* denoising approach with deep learning-based reconstruction

1. Image quality
  Representative images of the SN and LC from a 30-year-old healthy female volunteer are shown in Fig. 2. NEX1 + dDLR and NEX5 images visualize the SN and LC more clearly than NEX1 images. Results for SNR and CNR of healthy volunteers are presented in Fig. 3 and Table 2. *P* values are shown in Online Resource 4. SNR and CNR were significantly higher for NEX1 + dDLR than for NEX1 (*p* < 0.001) at the SN and LC. SNR and CNR from NEX1 + dDLR did not show any significant difference from those of NEX5. For patients with PD, SNR and CNR at the SN and LC were significantly higher for NEX1 + dDLR than for NEX1 (*p* < 0.001) (Table 2).

**Table 2:** Results of SNR and CNR from NEX1, NEX1 + dDLR and NEX5 of healthy volunteers and patients with PD

	Healthy volunteers			Patients with PD	
	NEX1	NEX1 + dDLR	NEX5	NEX1	NEX1 + dDLR
SNR_SCP	21.91 ± 3.38	36.29 ± 6.61*	33.42 ± 8.00*	20.47 ± 3.98	34.14 ± 7.78*
SNR_pons	17.07 ± 1.36	26.75 ± 2.84*	27.81 ± 2.62*	16.96 ± 1.31	26.58 ± 2.64*
CNR_SN	5.48 ± 0.83	8.92 ± 1.68*	8.65 ± 1.61*	3.82 ± 1.04	6.14 ± 1.91*
CNR_LC	4.50 ± 0.77	6.50 ± 1.11*	7.56 ± 1.39*	1.99 ± 0.32	2.91 ± 0.45

*Significantly higher than NEX1 (*p* < .001)

**Fig. 4:**
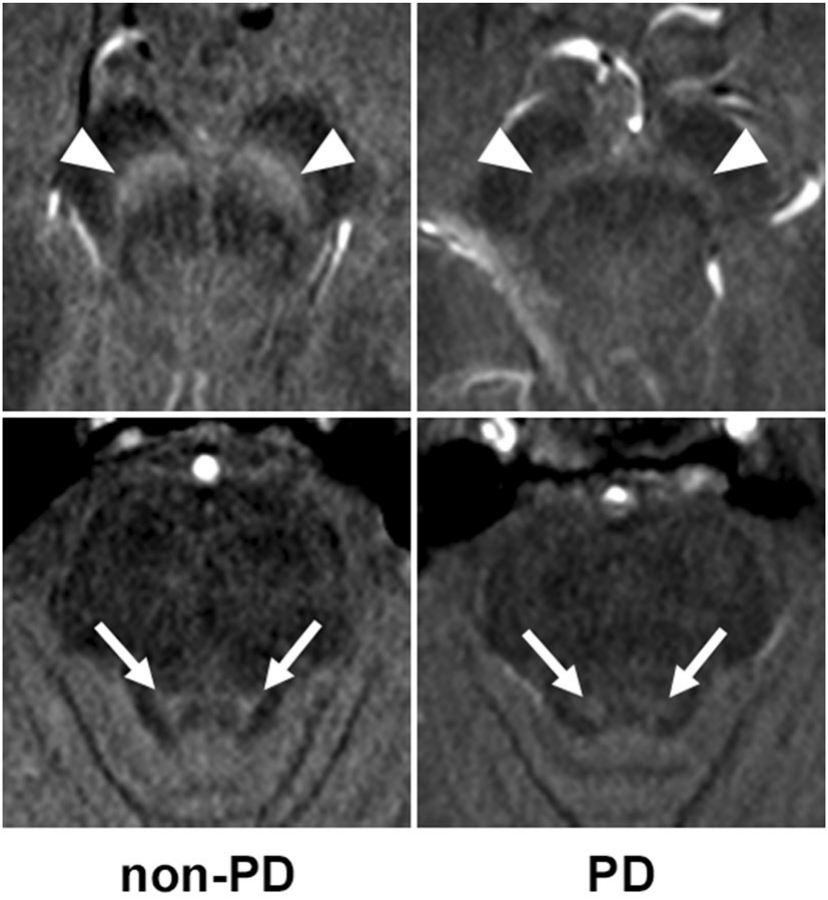
Images of the SN (arrowheads in the upper row) and LC (arrows in the lower row) from NEX1 + dDLR for a 73-year-old female non-PD patient (left column) and a 54-year-old female patient with PD (right column). *PD* Parkinson’s disease

**Fig. 5:**
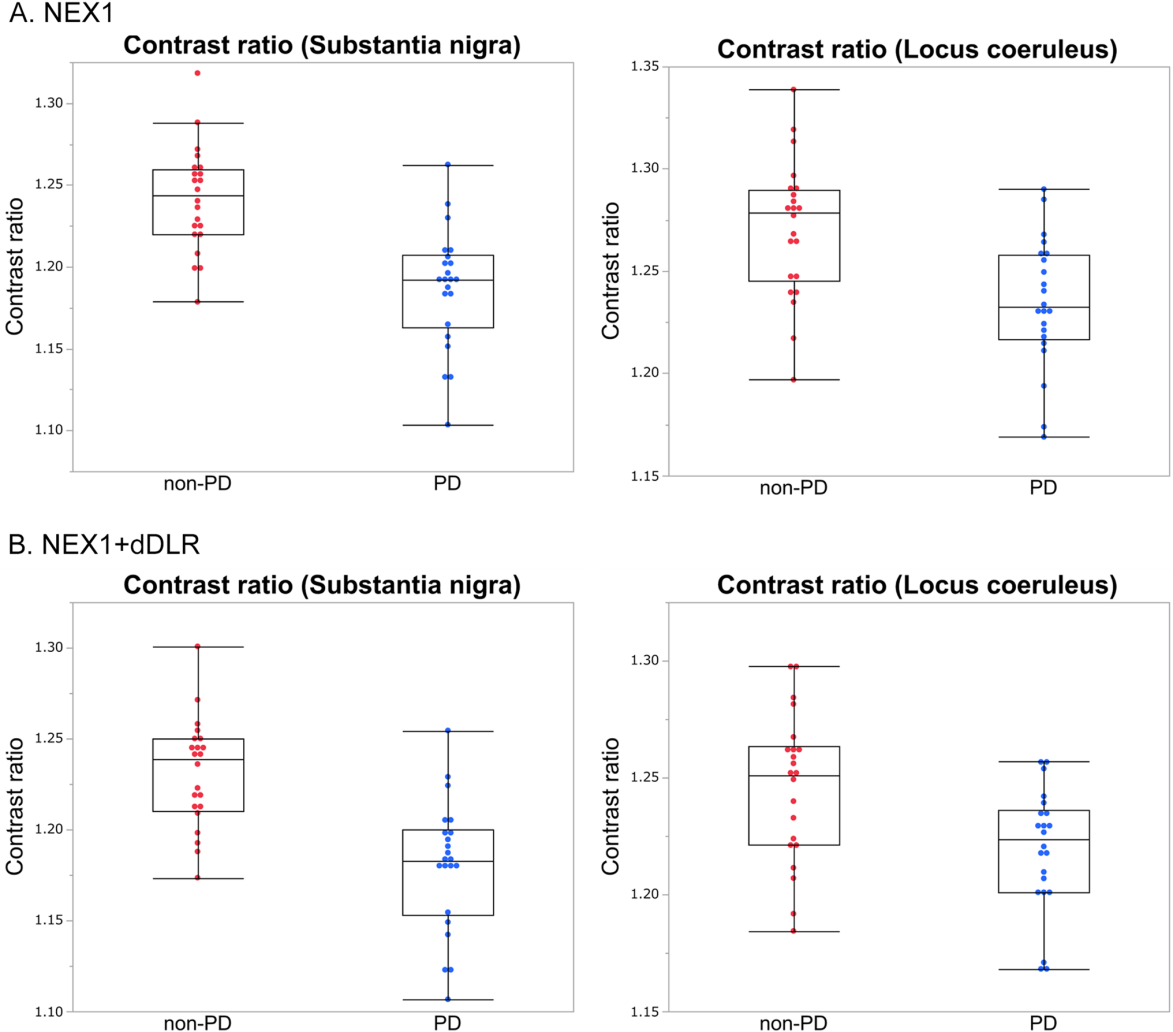
Box-and-whisker plots and scatter plots of contrast ratios for the substantia nigra and locus coeruleus in patients with PD and non-PD patients from NEX1 and NEX1 + dDLR. Contrast ratios of patients with PD were significantly lower than those of non-PD patients for both images. *PD* Parkinson’s disease

*NEX* number of excitations, *dDLR* denoising approach with deep learning-based reconstruction, *SNR_SCP* signal-to-noise ratio of the decussation of superior cerebellar peduncle, *SNR_pons* signal-to-noise ratio of the pons, *CNR_SN* contrast-to-noise ratio between the substantia nigra and decussation of superior cerebellar peduncle, *CNR_LC* contrast-to-noise ratio between the locus coeruleus and pons
  The results of qualitative assessment are shown in Online Resource 5. Scores for overall image quality, structural conspicuity and noise were significantly better for NEX5 among the three types of images (*p* < 0.01), and those for NEX1 + dDLR were significantly better than those of NEX1 (*p* < 0.001) for both the SN and LC. No significant differences in scores for artifacts were seen among the three images for both the SN and LC.
2. Diagnostic performance by contrast ratio
  Representative images of the SN and LC from a 73-year-old female non-PD patient and a 54-year-old female patient with PD are shown in Fig. 4. The SN and LC were visualized less clearly for the patient with PD compared to the non-PD patient. CR_SN and CR_LC from patients with PD were significantly decreased compared with those from non-PD patients in both NEX1 (CR_SN, 1.24 ± 0.03 for non-PD and 1.19 ± 0.04 for PD [*p* < 0.001]; CR_LC, 1.27 ± 0.03 for non-PD and 1.23 ± 0.03 for PD [*p* < 0.001]) and NEX1 + dDLR (CR_SN, 1.23 ± 0.03 for non-PD and 1.18 ± 0.04 for PD [*p* < 0.001]; CR_LC, 1.25 ± 0.03 for non-PD and 1.22 ± 0.03 for PD [*p* = 0.003]) (Fig. 5). The diagnostic performances of NEX1 and NEX1 + dDLR in differentiating PD from non-PD are presented in Table 3. AUCs of NEX1 + dDLR for CR_SN and CR_LC (CR_SN, 0.87 [95% confidence interval (CI), 0.73–0.95]; CR_LC, 0.75 [95%CI, 0.59–0.87]) were not significantly different from those of NEX1 (CR_SN, 0.87 [95%CI, 0.74–0.95]; CR_LC, 0.79 [95%CI, 0.64–0.90]). As for differentiation between healthy volunteers and patients with PD, AUCs of NEX1 + dDLR were 0.92 for CR_SN and 0.82 for CR_LC (Online Resource 6).

**Table 3:** Diagnostic performance of NEX1 and NEX1 + dDLR in differentiating PD from non-PD

		AUC	Optimal cutoff	Sensitivity	Specificity
CR_SN	NEX1	0.87 (0.74–0.95)	1.21	0.86 (0.65–0.97)	0.82 (0.60–0.95)
	NEX1 + dDLR	0.87 (0.73–0.95)	1.21	0.86 (0.65–0.97)	0.81 (0.60–0.95)
CR_LC	NEX1	0.79 (0.64–0.90)	1.26	0.82 (0.60–0.95)	0.68 (0.45–0.86)
	NEX1 + dDLR	0.75 (0.59–0.87)	1.24	0.82 (0.60–0.95)	0.64 (0.41–0.83)

95% confidence intervals are shown in parentheses

*NEX* number of excitations, *dDLR* denoising approach with deep learning-based reconstruction, *PD* Parkinson’s disease, *AUC*  area under the curve, *CR_SN* contrast ratio of the substantia nigra, *CR_LC* contrast ratio of the locus coeruleus
3. Diagnostic performance by visual assessment
  The results of diagnosis by visual assessment are shown in Table 4. Accuracy of each rater for NEX1 and NEX1 + dDLR was 0.45–0.59 and 0.59–0.64 for the SN, while 0.32–0.39 and 0.39–0.41 for the LC, respectively.

**Table 4:** Diagnostic performance by visual assessment of NEX1 and NEX1 + dDLR for differentiation between healthy volunteers and patients with PD

		Substantia Nigra						Locus Coeruleus					
		NEX1			NEX1 + dDLR			NEX1			NEX1 + dDLR		
		Rater 1	Rater 2	Rater 3	Rater 1	Rater 2	Rater 3	Rater 1	Rater 2	Rater 3	Rater 1	Rater 2	Rater 3
Number of cases	Correct diagnosis	26	26	20	28	26	26	17	16	14	17	18	17
	Difficult to diagnose	13	12	13	9	12	12	20	16	20	17	13	18
	Incorrect diagnosis	5	6	11	7	6	6	7	12	10	10	13	9
Accuracy	0.59	0.59	0.45	0.64	0.59	0.59	0.39	0.36	0.32	0.39	0.41	0.39	

*SN* substantia nigra, *LC* locus coeruleus

## Discussion

In this study, we applied dDLR to NM-MRI with an NEX of 1, which offers a much shorter scan time than conventional NM-MRI, and examined the resulting image quality and diagnostic performance. Image quality analyses showed approximately 1.5-fold improvement in SNR and CNR by applying dDLR to NEX1 images. The diagnostic performance using CR_SN and CR_LC in NEX1 + dDLR was comparable to that of NEX1. These results may suggest that NEX1 images are sufficient to differentiate between PD and non-PD patients and no apparent benefit for diagnostic performance by dDLR. However, diagnosis by visual assessment showed slight improvement of diagnostic performance by applying dDLR, which suggests the advantages of dDLR in diagnostic performance. Further tuning of dDLR would be expected to provide clinical merits in diagnosing PD. Our study showed a lower diagnostic capability of the LC for PD compared with that of the SN, which was consistent with previous studies [9, 31, 32]. The lower diagnostic performance of the LC than SN and the lower diagnostic performance of NEX1 + dDLR than NEX1 for the LC in our results may be because the LC is so small a structure that limited resolution of MR imaging can make it difficult to quantify the signal intensity accurately. Also, a previous study demonstrated that the optimal flip angle for LC imaging is different from that for SN [33]. Optimization of flip angle to increase contrast of LC may be required for stable measurement of LC and for taking full advantage of dDLR for LC images.

Several articles have already applied deep learning methods to MRI for diagnosing PD, such as for the creation of diagnostic biomarkers for PD [34], automatic segmentation of the SN on NM-MRI [35–37], and interpretation of nigrosome 1 on susceptibility map-weighted imaging [38]. However, to the best of our knowledge, no previous studies have applied deep learning-based denoising methods to NM-MRI. Our study demonstrated the utility of using dDLR for NM-MRI to reduce examination times without degrading image quality. Acquisition times for NEX5 and NEX1 are 15 min 58 s and 3 min 12 s, respectively; so, using NEX1 + dDLR instead of NEX5 would achieve a time reduction of around 12 min. This is quite advantageous, particularly for evaluating patients with PD who have tremors or involuntary movements. Furthermore, there is a possibility that denoising by dDLR can be beneficial for diagnosis by visual assessment. Considering that there have not been established criteria for visual diagnosis of PD by NM-MRI, further studies are required in the future.

Several limitations to this study should be considered. First, the number of participants was relatively small. Second, diagnostic performance using NEX5 images was not evaluated. This was because the scan time for NEX5 (15 min 58 s) was too long and uncomfortable for patients with PD, who suffer from tremors or involuntary movements with tolerate. Although we did not compare diagnostic performance between NEX1 + dDLR and NEX5, NEX1 + dDLR showed sufficiently high AUCs (0.87 for CR_SN, 0.75 for CR_LC). Third, age- and sex-matched healthy volunteers were not enrolled, because we recruited relatively young volunteers who could stay still during the scan of about 16 min. Diagnostic performance in this study was therefore evaluated between patients with PD and age- and sex-matched non-PD patients. Fourth, we used 2D NM-MRI in this study and we did not perform voxel-wise analysis as in previous papers [13]. Further investigations should be performed to assess the usefulness of dDLR in the application to 3D NM-MRI. Fifth, various 2D and 3D image sequences including turbo spin echo and GRE have been used for NM-MRI other than the 2D-GRE sequence we used. A future comprehensive study of NM-MRI using these sequences for healthy volunteers and patients with PD is required to determine the most appropriate NM-MRI. Lastly, our study used a vendor-supplied DLR algorithm to assess its clinical feasibility at only one institution. A multicenter study with various MRI scanners is therefore required.

In conclusion, 1-NEX NM-MRI with dDLR provided comparable image quality to 5-NEX NM-MRI, which represented the reference standard in this study. Our study demonstrated the potential of dDLR to reduce scan time of NM-MRI without degrading image quality. The diagnostic performance of 1-NEX NM-MRI using contrast ratio of the SN and that of LC was sufficiently good enough that dDLR did not further improve diagnostic accuracy in this study. However, the results of diagnosis by visual assessment suggest advantages of dDLR. Further tuning of dDLR would be expected not only to reduce scan time of NM-MRI without degrading image quality, but to provide clinical merits in diagnosing PD.

### Supplementary Information

Below is the link to the electronic supplementary material.

Supplementary file1 (DOCX 925 KB)
