# Supplementary material for: Denoising approach with deep learning-based reconstruction for neuromelanin-sensitive MRI: image quality and diagnostic performance
Source: Jpn J Radiol. 2023 May 31;41(11):1216–25. doi: 10.1007/s11604-023-01452-9 (PMC10613599; doi:10.1007/s11604-023-01452-9)
Supplement: Supplementary file 1 — Supplementary file1 (DOCX 925 KB) [file 11604_2023_1452_MOESM1_ESM.docx]

**Online Resource 1**





Convolutional neural network architecture of AiCE. The input image is convolved by the discrete cosine transformation in the first feature extraction layer to derive 49 components, which is divided into 48 high-frequency components and a zero-frequency component. A soft-shrinkage activation function is applied to 48 high-frequency components. Next, 48 high-frequency components undergo repeated convolution and soft shrinkage in the feature conversion layers. Finally, the denoised output image is generated by inverse discrete cosine transform convolution of both the output data from the feature conversion layers and the bypassed zero-frequency component. Note that the trapezoidal shape of the “Denoising level” expresses that the denoising level can be manually controlled.

DCT, discrete cosine transformation; IDCT, inverse discrete cosine transformation.

**Online Resource 2**


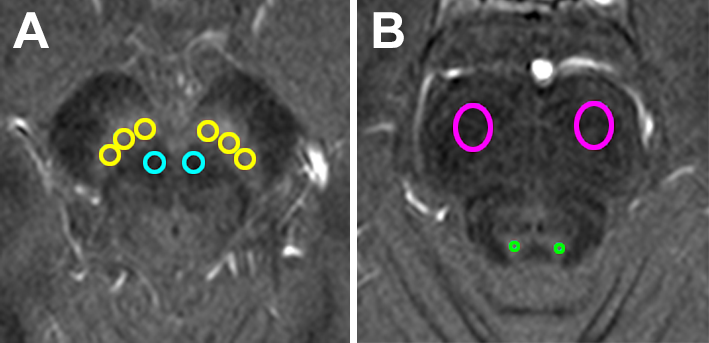


Images from NM-MRI at the SN (A) and LC (B) levels show examples of regions of interest for the SN (yellow circle), decussation of superior cerebellar peduncle (blue circle), pons (magenta oval) and LC (green circle).

**Online Resource 3. Image quality assessment using the 5-point Likert scale**

| Score | Overall image  quality | Artifacts | Structural conspicuity | Noise |
| --- | --- | --- | --- | --- |
| 1 | Nondiagnostic | Nondiagnostic | Unidentifiable | Remarkable noise |
| 2 | Substantial deficits in image quality | Substantial impact on diagnosis | Difficulties in delineating anatomic structures | Intermediate noise |
| 3 | Moderate image quality | Moderate impact on diagnosis | Seen with poorly defined edges | A little noise |
| 4 | Good image quality | Minimal impact on diagnosis | Well seen with poorly defined edges | Little noise |
| 5 | Excellent image quality | No artifact | Well-seen with well-defined edges | No noise |

**Online Resource 4. *P* values of comparisons of SNR and CNR between NEX1, NEX1 + dDLR and NEX5**

|  | SNR_SCP | SNR_pons | CNR_SN | CNR_LC |
| --- | --- | --- | --- | --- |
| NEX1 vs. NEX1 + dDLR | < .001 | < .001 | < .001 | < .001 |
| NEX1 vs. NEX5 | .02 | < .001 | .005 | < .001 |
| NEX1 + dDLR vs. NEX5 | 1.00 | 1.00 | 1.00 | .45 |

NEX, number of excitations; dDLR, denoising approach with deep learning-based reconstruction; SNR_SCP, signal-to-noise ratio of the decussation of superior cerebellar peduncle; SNR_pons, signal-to-noise ratio of the pons; CNR_SN, contrast-to-noise ratio between the substantia nigra and decussation of superior cerebellar peduncle; CNR_LC, contrast-to-noise ratio between the locus coeruleus and pons.

**Online Resource 5. Results of qualitative evaluation for NEX1, NEX1 + dDLR and NEX5**

|  |  | Overall image quality | Artifacts | Structural conspicuity | Noise |
| --- | --- | --- | --- | --- | --- |
| SN | NEX1 | 3 (2–3) | 5 (5) | 3 (3–4) | 2 (1–3) |
|  | NEX1 + dDLR | 4 (3–4) | 5 (5) | 4 (4–5) | 3 (3–4) |
|  | NEX5 | 5 (4–5) | 5 (4–5) | 5 (4–5) | 5 (3–5) |
| LC | NEX1 | 3 (2–3) | 5 (5) | 3 (3–4) | 2 (1–3) |
|  | NEX1 + dDLR | 4 (3–4) | 5 (5) | 4 (3–4) | 3.5 (3–4) |
|  | NEX5 | 5 (4–5) | 5 (5) | 5 (4–5) | 5 (4–5) |

The median and the range of scores are shown.

NEX, number of excitations; dDLR, denoising approach with deep learning-based reconstruction; SN, substantia nigra; LC, locus coeruleus.

**Online Resource 6. Diagnostic performance of NEX1 and NEX1 + dDLR for differentiation between healthy volunteers and patients with PD**

1. Contrast ratios of the substantia nigra and locus coeruleus in healthy volunteers and patients with PD

|  | | NEX1 | NEX1 + dDLR |
| --- | --- | --- | --- |
| CR_SN | Healthy volunteers | 1.24 ± 0.02 | 1.24 ± 0.03 |
|  | PD | 1.19 ± 0.04 | 1.18 ± 0.04 |
|  | *p* value | *p* < .001 | *p* < .001 |
| CR_LC | Healthy volunteers | 1.25 ± 0.03 | 1.25 ± 0.03 |
|  | PD | 1.23 ± 0.03 | 1.22 ± 0.03 |
|  | *p* value | *p* < .001 | *p* < .001 |

1. Results of diagnostic performance for differentiating patients with PD from healthy volunteers using contrast ratios of the substantia nigra and locus coeruleus

|  | | AUC | Optimal  cutoff | Sensitivity | Specificity |
| --- | --- | --- | --- | --- | --- |
| CR_SN | NEX1 | 0.91 (0.79–0.98) | 1.21 | 0.86 (0.65–0.97) | 0.95 (0.77–1.00) |
|  | NEX1 + dDLR | 0.92 (0.80–0.98) | 1.21 | 0.86 (0.65–0.97) | 0.95 (0.77–1.00) |
| CR_LC | NEX1 | 0.82 (0.68–0.92) | 1.26 | 0.82 (0.60–0.95) | 0.77 (0.55–0.92) |
|  | NEX1 + dDLR | 0.82 (0.68–0.92) | 1.24 | 0.86 (0.65–0.97) | 0.77 (0.55–0.92) |

Values in parentheses represent 95% confidence intervals.

NEX, number of excitations; dDLR, denoising approach with deep learning-based reconstruction; PD, Parkinson’s disease; CR_SN, contrast ratio of the substantia nigra; CR_LC, contrast ratio of the locus coeruleus; AUC, area under the curve
